# Maximizing Curcuminoid Extraction from *Curcuma aromatica* Salisb. Rhizomes via Environmentally Friendly Microwave-Assisted Extraction Technique Using Full Factorial Design

**DOI:** 10.1155/2024/4566123

**Published:** 2024-03-25

**Authors:** Jirapornchai Suksaeree, Chaowalit Monton

**Affiliations:** ^1^Department of Pharmaceutical Chemistry, College of Pharmacy, Rangsit University, Pathum Thani 12000, Thailand; ^2^Drug and Herbal Product Research and Development Center, College of Pharmacy, Rangsit University, Pathum Thani 12000, Thailand; ^3^Department of Pharmacognosy, College of Pharmacy, Rangsit University, Pathum Thani 12000, Thailand

## Abstract

*Curcuma aromatica* Salisb. contains a high content of curcuminoids, which can be utilized for cosmetic purposes. The objective of this study was to optimize the extraction conditions of *C. aromatica* rhizomes in castor oil to maximize curcuminoid content using a simple and environmentally friendly microwave-assisted extraction method. A 3^2^ full factorial design was employed, with two factors—microwave power and time—varying between 600-800 W and 30-90 s, respectively. Five responses were monitored, including extraction yield, bisdemethoxycurcumin, demethoxycurcumin, curcumin, and total curcuminoid contents. The results demonstrated that increasing microwave power and time led to an increase in all five responses. The optimal condition, which simultaneously maximized extraction yield and total curcuminoid content, was achieved at a microwave power of 800 W for 90 s. This condition resulted in an extraction yield of 71.020%, bisdemethoxycurcumin content of 0.036%, demethoxycurcumin content of 0.210%, curcumin content of 0.080%, and total curcuminoid content of 0.326%. The computer program accurately predicted the results with a percentage error of less than 2%. Stability data revealed that the total curcuminoid content remained stable with a percentage remaining above 90% when stored at 4°C, 30°C ± 75%RH, and 40°*C* ± 75%RH for three months. In summary, this study successfully applied a full factorial design to maximize curcuminoid extraction from *C. aromatica* rhizomes using an environmentally friendly microwave-assisted extraction method for cosmetic purposes.

## 1. Introduction

*Curcuma aromatica* Salisb., a member of the Zingiberaceae family, exhibits a wide range of beneficial activities, including anticancer, anti-inflammatory, antimelanogenic, antioxidant, antinephrotoxic, antiplatelet, antitumor, antitussive, mosquito repellent, and wound healing effects [1]. It possesses skin-nourishing properties through its antioxidant activity. The rhizomes of *C. aromatica* contain curcuminoids, including curcumin (CUR), demethoxycurcumin (DMC), and bisdemethoxycurcumin (BDMC), as the major chemical constituents. Among these curcuminoids, CUR is the predominant compound, and its antioxidant mechanism has been extensively studied [2, 3]. CUR has the ability to protect biomembranes by scavenging reactive free radicals, thereby preventing peroxidative damage. Another antioxidant mechanism of CUR has been elucidated, involving its degradation into four compounds: trans-6-4′-hydroxy-3′-methoxyphenyl-2,4-dioxo-5-hexanal, ferulic acid, feruloylmethane, and vanillin [4]. This degradation process occurs within thirty minutes under alkaline pH conditions [4]. Notably, both ferulic acid and vanillin also exhibit antioxidant activity [4]. Several in vitro assays have confirmed the strong antioxidant properties of CUR [5]. Additionally, DMC and BDMC have also been found to possess antioxidant activity [6].

Extraction plays a crucial role in the isolation, chemical analysis, and assessment of the biological and pharmacological properties of plant compounds. The extraction process and techniques employed are vital in obtaining a substantial quantity of desired active compounds from plants while preventing the degradation of sensitive compounds [7]. The extraction techniques employed significantly impact the yield and content of active compounds, as well as their activities [8]. Therefore, the careful selection of extraction conditions is a critical stage that must be given due consideration. Traditional extraction techniques were used for extraction of *C. aromatica* rhizomes, for example, maceration [9], reflux [10, 11], and the Soxhlet extraction [12, 13]. The traditional extraction techniques have some major drawbacks, including taking a long time and requiring large solvent volumes. In addition, they are energy-consuming, resulting in methods that are not environmentally friendly [14]. Recently, several modern extraction techniques recognized as environmentally friendly have also been performed, such as ultrasound-assisted extraction [15] and supercritical fluid extraction [13]. However, ultrasound-assisted extraction requires a significant amount of time, and supercritical fluid extraction involves higher production costs compared to microwave-assisted extraction (MAE) [14]. Therefore, MAE is one of the interesting techniques for extracting curcuminoids from *C. aromatica* rhizomes.

Microwaves, which belong to the electromagnetic radiation category, encompass frequencies ranging from 0.3 to 300 GHz. When it comes to extracting natural products, microwaves are commonly utilized within the 2.5–75 GHz range. The effectiveness of microwave energy in the extraction process primarily relies on factors like solvent concentration, plant composition, extraction duration, and the intensity of microwave radiation [16]. MAE has gained extensive recognition and adoption in diverse fields such as pharmacy, chemistry, medicine, and material sciences. MAE is favored as an extraction technique due to its numerous advantages over other methods, including reduced costs and time, lower solvent and energy consumption, and lower emission of carbon dioxide. Consequently, it is regarded as an environmentally friendly extraction technique [17]. It has been utilized in various studies for herbal drying [18, 19] and herbal extraction processes [20–22].

In the process of extracting active compounds from plants, scientists purposely adjust certain factors in order to observe how they affect specific outcomes in an experiment. To effectively organize and carry out these experiments and gather accurate data for analysis, researchers employ the design of experiment (DOE) method [23]. Traditional experiments frequently depend on the trial-and-error approach, which can be ineffective and lack organization, particularly when conducted without prior knowledge of the subject matter. Moreover, the one factor at a time (OFAT) method is employed, wherein one factor is altered, and the corresponding response is measured before moving on to another factor. However, this method seldom identifies the optimal combination of conditions involving multiple factors. In contrast, the application of the DOE can overcome these challenges [24]. DOE offers various advantages over traditional OFAT methods, including time, budget, and resource savings due to smaller run numbers, the ability to identify interactions between factors, and the characterization of response surfaces [25, 26]. Furthermore, DOE allows the use of statistical models to predict the effects of multiple factors simultaneously [24].

Previously, it was proven that castor oil can extract the highest content of curcuminoids from organic *C. longa* rhizomes using MAE. Moreover, it demonstrated better preservation of curcuminoids during stability tests compared to coconut oil and sesame oil [20]. Therefore, in this study, castor oil was also utilized to extract curcuminoids from *C. aromatica* rhizomes. The main objective was to optimize the extraction conditions of *C. aromatica* rhizomes in castor oil, maximizing the curcuminoid content through a simple and environmentally friendly MAE method. Additionally, the extracted solution obtained under the optimal extraction conditions was subjected to a stability test to evaluate its stability properties.

## 2. Materials and Methods

### 2.1. Materials

Rhizomes of *C. aromatica* were obtained from Charoensuk Osod, Nakhon Pathom Province, Thailand. Three standard curcuminoids including BDMC, DMC, and CUR were purchased from Chengdu Biopurify Phytochemicals Ltd., China. Acetonitrile and methanol (HPLC grade) were purchased from Fisher Chemical, Loughborough, UK. Acetic acid (AR grade) was purchased from Carlo Erba Reagents, Italy. Castor oil was purchased from Krungthepchemi, Thailand.

### 2.2. Plant Sample Preparation

The rhizomes of *C. aromatica* were examined by plant taxonomist Ajarn Nirun Vipunngeun to ensure accurate species identification. They were assigned the code CM-CA001-1-11-2020, and the corresponding voucher specimens were deposited at the Drug and Herbal Product Research and Development Center, College of Pharmacy, Rangsit University. The rhizomes were cleaned with tap water and subsequently air-dried. They were then sliced and subjected to drying in a hot-air oven at 60°C for 6 h. After drying, the rhizomes were pulverized and passed through a 60-mesh sieve.

### 2.3. Experimental Design Using Full Factorial Design

The MAE conditions were established using a 3^2^ full factorial design, specifically a 2-factor 3-level full factorial design. The two factors, microwave power and time, were coded as *X*_1_ and *X*_2_, respectively. Microwave power was varied at levels of 600, 700, and 800 W, while microwave time was varied at levels of 30, 60, and 90 s. A total of nine conditions were obtained, and the center of the design was replicated three times, resulting in a total of 11 conditions.

Five responses were monitored, namely, extraction yield, BDMC content, DMC content, CUR content, and total curcuminoid content, which were coded as *Y*_1_ to *Y*_5_, respectively. After conducting the extractions under the specified conditions, all response values were analyzed using Design-Expert® version 11.0. Response surfaces were constructed for each response. The coded equation; plots showing the relationship between predicted values and actual values with *R*^2^, adjusted *R*^2^, and predicted *R*^2^; perturbation plots; and analysis of variance (ANOVA) for the quadratic model were reported for each response.

**Figure 1:**
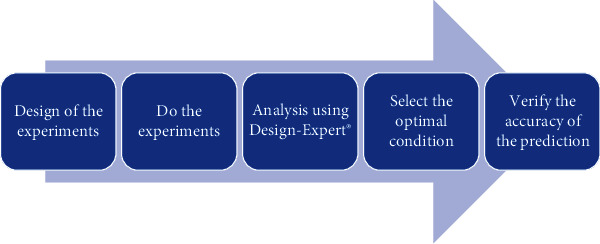
Workflow for the full factorial experiment design.

The optimal condition, determined through the desirability function, was chosen to maximize both the extraction yield and total curcuminoid content. This selection was made for the purpose of conducting a verification step to confirm the accuracy of the predictions generated by the Design-Expert® program. Workflow for the full factorial experiment design is shown in Figure 1.

### 2.4. Microwave-Assisted Extraction

*C. aromatica* rhizome powder (6 g) was placed in a 250 mL Erlenmeyer flask, added 20 g of castor oil, and stirred using a stirring rod for 10 s before undergoing microwave treatment. The microwaving was performed using a microwave oven model MS23F300EEK (Samsung, Bangkok, Thailand) with specific microwave power and time settings as detailed in Table 1. Each condition was replicated three times.

After a predetermined time, the mixture was filtered using a vacuum pump filtration system through a 3-layer polyester cloth. The weight of the resulting extract was measured, and the extraction yield was calculated by comparing it to the initial weight of *C. aromatica* rhizome powder and castor oil (26 g). The obtained extracts were further analyzed for their curcuminoid content using high-performance liquid chromatography (HPLC).

### 2.5. Sample Preparation for HPLC Analysis

The extract weighing 100 mg was transferred into a 10 mL volumetric flask and dissolved in methanol. The solution was then adjusted to the desired volume. Subsequently, it was filtered, and the contents of three curcuminoids were analyzed using their respective calibration curves. The combined value of the curcuminoid contents was reported as the total curcuminoid content.

The analysis was performed using the Agilent 1260 Infinity HPLC instrument (Agilent Technologies, USA). For the separation, an ACE Generix column with dimensions of 150 mm in length, 4.6 mm in internal diameter, and 5 *μ*m in particle size was utilized. An isocratic system consisting of a mixture of acetonitrile (55% v/v) and a 1% acetic acid aqueous solution (45% v/v) was employed at a flow rate of 1 mL/min. The column temperature was maintained at 30°C. A 10 *μ*L injection volume was used. Detection of the analysis response was conducted at a wavelength of 425 nm using a photodiode array detector [27].

### 2.6. Stability Test

The extract obtained under the optimal extraction conditions was stored in airtight containers and protected from light. It was kept in a refrigerator at 4°C as well as in a climate chamber (Memmert, Germany) at conditions of 30°C/75%RH and 40°C/75%RH for a period of three months. After the storage period, the remaining curcuminoid content was analyzed using HPLC, and the results were compared to the initial time point to assess any changes.

### 2.7. Statistical Analysis

Differences in the content of each curcuminoid as well as the total curcuminoid content were analyzed using analysis of variance (ANOVA) by SPSS software version 22 (IBM, USA). Subsequently, post hoc analysis was conducted using the LSD method. A significance level of *p* < 0.05 was used, indicating statistical significance at a 95% confidence interval.

## 3. Results and Discussion

### 3.1. Effect of Microwave Power and Time on Extraction Yield and Curcuminoid Contents

**Figure 2:**
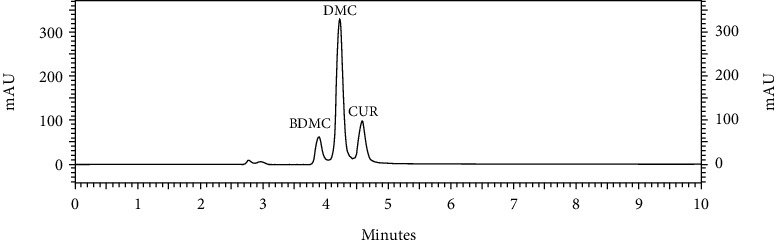
HPLC chromatogram of extract of *C. aromatica* rhizomes in castor oil (10 mg/mL).

The MAE conditions were designed using a 3^2^ full factorial design. Microwave power (*X*_1_) and time (*X*_2_) were varied to evaluate their effects on extraction yield (*Y*_1_), individual curcuminoid content (*Y*_2_ to *Y*_4_), and total curcuminoid content (*Y*_5_).  Figure 2 shows the HPLC chromatogram of the extract of *C. aromatica* rhizomes in castor oil. The extract primarily contained DMC, followed by CUR and BDMC, respectively. These three curcuminoids were previously reported in the literature. In that study, *C. aromatica* rhizome powder extracted in absolute ethanol using an ultrasound-assisted extraction method contained 0.43% DMC, 0.09% BDMC, and 0.08% CUR, with a total curcuminoid content of 0.6% [27]. These values seem higher than what was found in the present work. Sometimes, CUR can be reported as the major curcuminoid instead of DMC [28, 29]. A study reported that *C. aromatica* methanolic extract contained 0.11% CUR with no BDMC and no DMC [30]. This finding is comparable to another study that reported *C. aromatica* to contain CUR at 0.05-0.1% of the dried weight [31]. However, it should be noted that the previous work reported the dried extract of *C. aromatica* rhizomes, while the present work calculates the curcuminoid content based on the extract in castor oil. Therefore, the curcuminoids were diluted with castor oil. The authors highlighted that this extract claims to be a ready-to-use ingredient that can be incorporated into cosmetic formulations without requiring further dissolution.

The response values obtained from the design are shown in Table 1. The coded equations generated from the model conditions are presented below as

(1) $$\begin{array}{c} Y_{1}=65.10+3.64X_{1}+2.94X_{2}, \end{array}$$

(2) $$\begin{array}{c} Y_{2}=0.0319+0.0007X_{1}+0.0041X_{2}+0.0007X_{1}X_{2}-0.0014X_{2}^{2}, \end{array}$$

(3) $$\begin{array}{c} Y_{3}=0.1946+0.0031X_{1}+0.0207X_{2}+0.0034X_{1}X_{2}+0.0006X_{1}^{2}-0.0087X_{2}^{2}, \end{array}$$

(4) $$\begin{array}{c} Y_{4}=0.0740+0.0008X_{1}+0.0073X_{2}+0.0017X_{1}X_{2}+0.0001X_{1}^{2}-0.0032X_{2}^{2}, \end{array}$$

(5) $$\begin{array}{c} Y_{5}=0.3004+0.0045X_{1}+0.0321X_{2}+0.0059X_{1}X_{2}+0.0006X_{1}^{2}-0.0132X_{2}^{2}. \end{array}$$

The coefficient values of the factors for each response revealed that microwave time (*X*_2_) had the greatest effect compared to microwave power (*X*_1_) on each response, except *Y*_1_. All terms, except the quadratic term of *X*_2_, had a positive effect on all responses, while only the quadratic term of *X*_2_ had a negative effect on the responses.

**Figure 3:**
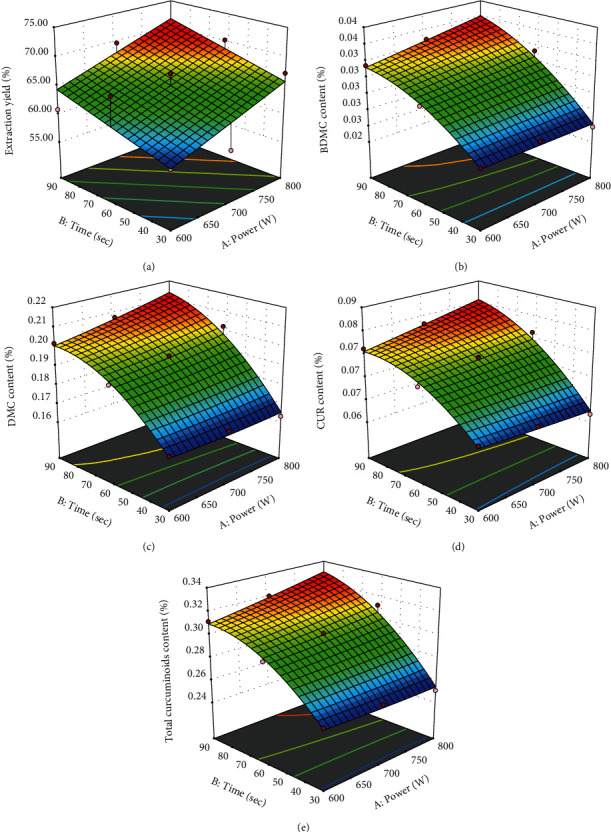
Response surfaces of (a) extraction yield, (b) BDMC content, (c) DMC content, (d) CUR content, and (e) total curcuminoid content.

The three-dimensional response surfaces of each response are shown in Figure 3. They indicated that increasing microwave power and time increased extraction yield, individual curcuminoid, and total curcuminoid content. The highest extraction yields as well as individual and total curcuminoid contents could be observed at high microwave power and time.

In a prior investigation, researchers utilized the MAE method to extract curcuminoids from *C. longa* rhizome, employing coconut oil as a solvent. Employing the Box-Behnken design, they scrutinized the impact of solid content, microwave time, and irradiation cycle on curcuminoid content, finding that low solid content, extended microwave time, and a high number of irradiation cycles yielded the highest extraction efficiency. Similarly, the highest individual and total curcuminoid contents were achieved with high solid content, prolonged microwave time, and an increased number of irradiation cycles [20]. Similar to previous work, our study found that longer microwave treatments led to higher levels of curcuminoids, alongside increased extraction yield.

**Figure 4:**
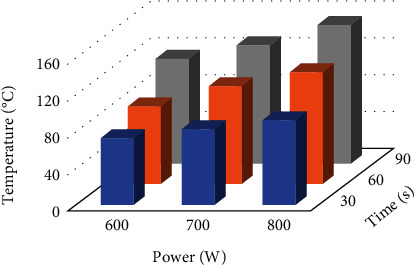
The observed temperature change as a function of applied microwave conditions.

The effects of microwave power and time were also assessed in the extraction of astilbin from *Lysiphyllum strychnifolium* (Craib) A. Schmitz stem for its anti-inflammatory properties. It was found that increasing microwave power led to higher extraction yield and astilbin yield, while increasing the microwave time decreased the astilbin yield in the extract [32]. However, a negative effect of a factor on thermosensitive bioactive compounds such as astilbin could be observed [33]. This result suggests that prolonged exposure to heat can lead to the degradation of certain bioactive compounds. Another work reported that increasing microwave power and time can enhance the extraction yield and total phenolic content of a mixture containing *Caesalpinia sappan* L., *Hibiscus sabdariffa* L., and *Clitoria ternatea* L. This study suggests that the phenolic compounds present in these plants are relatively stable. However, regarding the total flavonoid content, there was an optimal value observed. Increasing microwave time initially increased the total flavonoid content until reaching a maximum value. However, further prolonging the microwave time could result in the degradation of certain flavonoids [34]. In another study, slight differences were observed. Although increasing microwave power and time led to an increase in the extraction yield, microwave time did not significantly affect the total phenolic content. On the other hand, microwave power exhibited an optimal value for total phenolic content. Increasing the microwave power initially increased the total phenolic content until reaching a maximum value. However, further increasing the microwave power could lead to the degradation of certain phenolic compounds [21]. The data presented above indicate the need for optimization of the extraction process for each plant in order to maximize both the extraction yield and the content of bioactive compounds. The authors mentioned that the microwave power used significantly impacts the extraction process through its influence on temperature (Figure 4). However, due to limitations of the instrument used, accurate temperature control was not possible in this work. In the case of the curcuminoids observed in this present study, they appeared to remain stable even when the temperature reached approximately 150°C (as indicated by the observed temperature changes as a function of applied microwave conditions, shown in Figure 4), even under the most extreme conditions such as a microwave power of 800 W for 90 s. This work highlighted the advantage of MAE over other heat-related extraction techniques. During MAE, the *C. aromatica* rhizome powder was exposed to heat for a short duration. Consequently, curcuminoids could be preserved from degradation.

**Figure 5:**
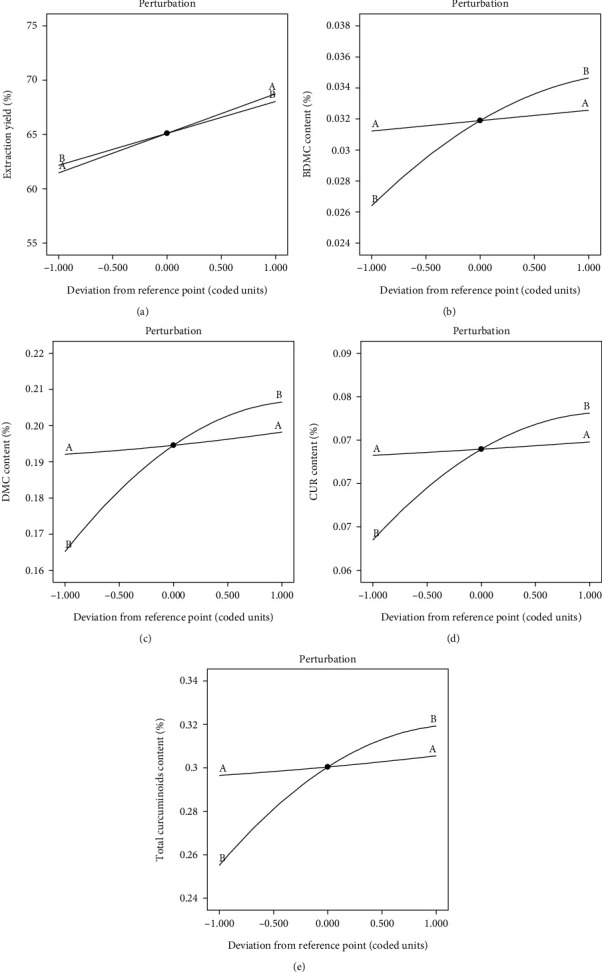
Perturbation plots of (a) extraction yield, (b) BDMC content, (c) DMC content, (d) CUR content, and (e) total curcuminoid content. (A) is microwave power (*X*_1_), and (B) is microwave time (*X*_2_).

The perturbation plots for each response are presented in Figure 5 to demonstrate the effects of all the factors at a specific point in the design space. Each response was plotted by varying a single factor across its range while keeping all other factors constant.  Figure 5(a) illustrates that microwave power had a greater effect on extraction yield compared to microwave time. Figures 5(b)–5(e) reveal that microwave time had a greater effect on curcuminoid content. The data from the perturbation plots are consistent with the coded equations presented in Equations (1)–(5).

**Figure 6:**
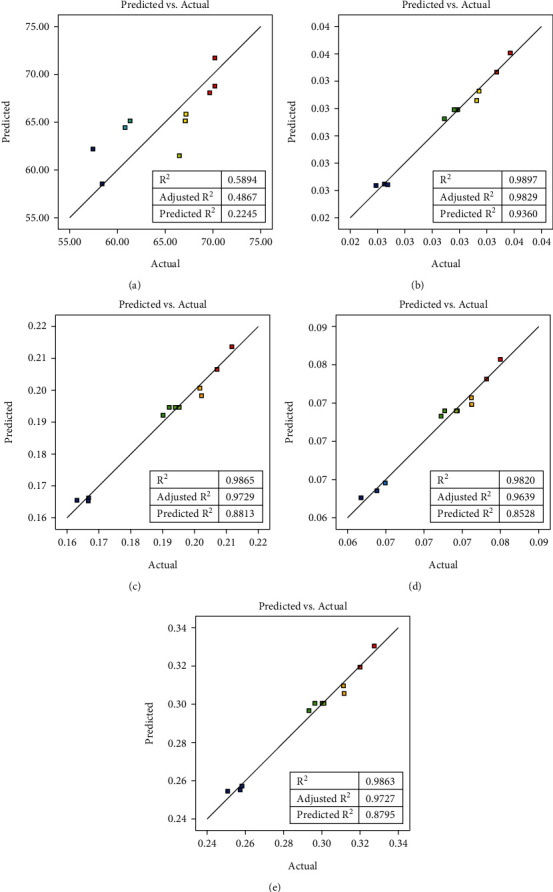
Predicted vs. actual value plots of (a) extraction yield, (b) BDMC content, (c) DMC content, (d) CUR content, and (e) total curcuminoid content.

The plots between predicted and actual values are shown in Figure 6. All *R*^2^ values for each response were high, except *R*^2^ for extraction yield; the difference between the predicted *R*^2^ and the adjusted *R*^2^ values was less than 0.2 indicating that they were in reasonable agreement [35]. The above data indicated that the model was fitted. The low *R*^2^ value for the extraction yield could be attributed to variations in the filtration step, where the castor oil may have been absorbed by the *C. aromatica* rhizome powder. This absorption could lead to variations in the obtained extraction yield.

The ANOVA data for the model of each response are shown in Table 2. They revealed that the model was significant while lack of fit was not significant, indicating that the model was fitted. Microwave power (*X*_1_) was a significant term for extraction yield, BDMC content, and DMC content. Microwave time (*X*_2_) was a significant term for BDMC, DMC, CUR, and total curcuminoid contents. *X*_1_*X*_2_ was a significant term for BDMC, CUR, and total curcuminoid contents. *X*_2_^2^ was a significant term for BDMC, DMC, CUR, and total curcuminoid contents.

### 3.2. Optimal Extraction Condition of MAE

The optimal condition, which demonstrated a high extraction yield and total curcuminoid content simultaneously based on the desirability function, was selected for the verification step to validate the accuracy of the predictions generated by the Design-Expert® program. The optimal condition, determined as a microwave power of 800 W for 90 s with a desirability value of 1.000, was utilized to extract the *C. aromatica* rhizome powder once again. The results demonstrated that the experimental values closely matched the predicted values. The percent error was very close to 0%, indicating the accuracy of the prediction made by the Design-Expert® program (Table 3).

### 3.3. Stability of Extract Obtained from the Optimal Condition

**Figure 7:**
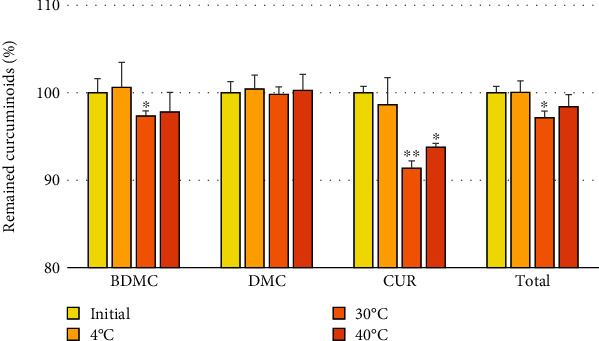
The stability of the *C. aromatica* rhizome powder extract in castor oil at 4°C, 30°C/75%RH, and 40°C/75%RH for three months. The differences in the contents of curcuminoids were compared to the initial time point. Significant values were indicated by an asterisk (^∗^): ^∗^*p* < 0.01 and ^∗∗^*p* < 0.001.

The extract of *C. aromatica* rhizome powder obtained using the optimal MAE condition was subjected to stability evaluation by storing it in a refrigerator (4°C) and a climate chamber (30°C/75%RH and 40°C/75%RH) for three months to assess the remaining curcuminoid content. The stability data are presented in Figure 7. The results indicated that the individual and total curcuminoid contents exhibited good stability when stored in the refrigerator at 4°C. However, when stored at 30°C/75%RH, there was a significant decrease in the contents of BDMC, CUR, and total curcuminoids. Furthermore, CUR also showed a significant decrease when stored at 40°C/75%RH. It has been previously reported that CUR has lower stability compared to BDMC and DMC [20]. While curcuminoids are often considered heat-resistant, their breakdown increases with higher temperatures and longer heating times [36]. In this case, 30°C and 40°C seemed low temperatures that could not lead to instability of curcuminoids. Therefore, decreasing curcuminoid content would participate in additional and antioxidative reactions. Nevertheless, the individual and total curcuminoid contents remained above 90%, indicating that the product remained stable and suitable for use without any significant deterioration [37].

The stability data of the *C. aromatica* rhizome powder extract in castor oil were found to be comparable to the stability of *C. longa* rhizome powder extract in castor oil, as reported previously. Castor oil demonstrated better preservation of curcuminoid stability compared to other oils such as coconut oil and sesame oil. The individual and total curcuminoid contents did not show significant alterations when the extract was stored at 30°C/75%RH and 40°C/75%RH for three months [20].

## 4. Conclusions

This study successfully optimized the extraction conditions for maximizing curcuminoid content from *C. aromatica* rhizomes using an environmentally friendly MAE method. The optimal condition was determined as a microwave power of 800 W for 90 s. The accuracy of the results was confirmed by the computer program, which exhibited a low percentage error. Furthermore, stability testing revealed that the total curcuminoid content remained stable, with a percentage remaining above 90% when stored under different temperature and humidity conditions for three months. Overall, this study provides valuable insights into the optimization of curcuminoid extraction from *C. aromatica* rhizomes for cosmetic applications. The environmentally friendly MAE technique offers a sustainable and efficient method for obtaining high curcuminoid yields. Further research can explore the application of optimized extraction conditions in large-scale production for commercial purposes.

## Figures and Tables

**Table 1:** Factors and responses of the 3^2^ full factorial design.

Run	Factors		Responses				
	Power (W)	Time (s)	Extraction yield (%)	BDMC content (%)	DMC content (%)	CUR content (%)	Total curcuminoid content (%)
1	600	30	58.42 ± 7.24	0.027 ± 0.006	0.167 ± 0.035	0.065 ± 0.013	0.258 ± 0.054
2	700	30	57.46 ± 3.27	0.027 ± 0.003	0.167 ± 0.012	0.064 ± 0.005	0.257 ± 0.019
3	800	30	67.19 ± 5.11	0.026 ± 0.002	0.163 ± 0.008	0.062 ± 0.003	0.251 ± 0.012
4	600	60	66.51 ± 7.05	0.031 ± 0.001	0.190 ± 0.001	0.072 ± 0.001	0.293 ± 0.003
5^∗^	700	60	67.11 ± 4.35	0.032 ± 0.000	0.194 ± 0.004	0.074 ± 0.002	0.300 ± 0.006
6^∗^	700	60	67.14 ± 5.33	0.032 ± 0.000	0.195 ± 0.003	0.074 ± 0.001	0.301 ± 0.005
7^∗^	700	60	61.35 ± 5.14	0.032 ± 0.000	0.192 ± 0.003	0.073 ± 0.002	0.297 ± 0.005
8	800	60	70.22 ± 4.46	0.033 ± 0.001	0.202 ± 0.003	0.076 ± 0.002	0.312 ± 0.005
9	600	90	60.83 ± 3.38	0.033 ± 0.000	0.202 ± 0.002	0.076 ± 0.001	0.311 ± 0.002
10	700	90	69.68 ± 3.99	0.035 ± 0.001	0.207 ± 0.002	0.078 ± 0.002	0.320 ± 0.004
11	800	90	70.21 ± 5.85	0.036 ± 0.001	0.212 ± 0.003	0.080 ± 0.001	0.328 ± 0.005

^∗^ Replicate conditions at the center point of the experimental design.

**Table 2:** ANOVA for the linear model of extraction yield, the reduced quadratic model for BDMC content, and the quadratic models for DMC, CUR, and total curcuminoid contents.

Variable	Sum of squares	df	Mean square	*F*-value	*p* value	Significance
*Extraction yield*						
Model	131.58	2	65.79	5.74	0.0284	Significant
*X*_1_: power	79.69	1	79.69	6.95	0.0298	Significant
*X*_2_: time	51.89	1	51.89	4.53	0.0660	Not significant
Lack of fit	69.42	6	11.57	1.04	0.5659	Not significant
Pure error	22.26	2	11.13			
Cor total	223.26	10				
*BDMC content*						
Model	0.0001	4	0.0000	144.53	<0.0001	Significant
*X*_1_: power	2.700 × 10^−6^	1	2.700 × 10^−6^	13.94	0.0097	Significant
*X*_2_: time	0.0001	1	0.0001	526.76	<0.0001	Significant
*X*_1_ *X*_2_	2.115 × 10^−6^	1	2.115 × 10^−6^	10.92	0.0163	Significant
*X*_2_^2^	5.136 × 10^−6^	1	5.136 × 10^−6^	26.51	0.0021	Significant
Lack of fit	1.131 × 10^−6^	4	2.827 × 10^−7^	17.93	0.0535	Not significant
Pure error	3.153 × 10^−8^	2	1.577 × 10^−8^			
Cor total	0.0001	10				
*DMC content*						
Model	0.0029	5	0.0006	72.83	0.0001	Significant
*X*_1_: power	0.0001	1	0.0001	7.19	0.0438	Significant
*X*_2_: time	0.0026	1	0.0026	325.57	<0.0001	Significant
*X*_1_ *X*_2_	0.0000	1	0.0000	5.99	0.0581	Not significant
*X*_1_^2^	9.145 × 10^−7^	1	9.145 × 10^−7^	0.1163	0.7469	Not significant
*X*_2_^2^	0.0002	1	0.0002	24.38	0.0043	Significant
Lack of fit	0.0000	3	0.0000	4.72	0.1798	Not significant
Pure error	4.866 × 10^−6^	2	2.433 × 10^−6^			
Cor total	0.0029	10				
*CUR content*						
Model	0.0004	5	0.0001	54.41	0.0002	Significant
*X*_1_: power	3.549 × 10^−6^	1	3.549 × 10^−6^	2.66	0.1637	Not significant
*X*_2_: time	0.0003	1	0.0003	240.22	<0.0001	Significant
*X*_1_ *X*_2_	0.0000	1	0.0000	9.05	0.0298	Significant
*X*_1_^2^	9.778 × 10^−9^	1	9.778 × 10^−9^	0.0073	0.9351	Not significant
*X*_2_^2^	0.0000	1	0.0000	18.89	0.0074	Significant
Lack of fit	4.962 × 10^−6^	3	1.654 × 10^−6^	1.94	0.3577	Not significant
Pure error	1.703 × 10^−6^	2	8.516 × 10^−7^			
Cor total	0.0004	10				
*Total curcuminoid content*						
Model	0.0069	5	0.0014	72.23	0.0001	Significant
*X*_1_: power	0.0001	1	0.0001	6.38	0.0528	Not significant
*X*_2_: time	0.0062	1	0.0062	323.16	<0.0001	Significant
*X*_1_ *X*_2_	0.0001	1	0.0001	7.28	0.0429	Significant
*X*_1_^2^	1.042 × 10^−6^	1	1.042 × 10^−6^	0.0545	0.8246	Not significant
*X*_2_^2^	0.0004	1	0.0004	23.16	0.0048	Significant
Lack of fit	0.0001	3	0.0000	4.39	0.1910	Not significant
Pure error	0.0000	2	6.298 × 10^−6^			
Cor total	0.0070	10				

**Table 3:** The verification data presented the predicted values, experimental values, and percent error of the predictions.

Responses	Predicted values	Experimental values	Error (%)^∗^
Extraction yield (%)	71.686	71.020 ± 4.928	-0.938
BDMC content (%)	0.036	0.036 ± 0.000	-0.000
DMC content (%)	0.214	0.210 ± 0.001	-1.905
CUR content (%)	0.081	0.080 ± 0.001	-1.250
Total curcuminoid content (%)	0.330	0.326 ± 0.002	-1.227

^∗^Error (%) = (experimental value–predicted value) × 100/experimental value.

## Data Availability

The data used to support the findings of this study are available from the corresponding author upon request.
